# Hydrothermal Processing of *Clarias gariepinus* (Burchell, 1822) Filets: Insights on the Nutritive Value and Organoleptic Parameters

**DOI:** 10.3390/vetsci7030133

**Published:** 2020-09-11

**Authors:** Victor Tosin Okomoda, Lateef Oloyede Tiamiyu, Amighty Olorunpelumi Ricketts, Sunday Abraham Oladimeji, Austine Agbara, Mhd Ikhwanuddin, Korede Isaiah Alabi, Ambok Bolong Abol-Munafi

**Affiliations:** 1 Department of Fisheries and Aquaculture, College of Forestry and Fisheries, University of Agriculture Makurdi, 2373 Makurdi, Nigeria; amightyricketts@yahoo.com.au (A.O.R.); agbaraa@yahoo.com (A.A.); 2 Institute of Tropical Aquaculture and Fisheries (AKUATROP), Universiti Malaysia Terengganu, 21030 Kuala Nerus, Terengganu, Malaysia; ikhwanuddin@umt.edu.my; 3 Department of Aquaculture and Fisheries, Faculty of Agriculture, University of Ilorin, PMB 1515 Ilorin, Nigeria; lottiamiyu@gmail.com; 4 Agricultural Department, National Biotechnology Development Agency (NABDA), 5118 Abuja, Nigeria; sunnybleek2013@gmail.com; 5 Department of Agricultural Extension and Management, Federal College of Forestry, 2019 Jos. Plateau, Nigeria; korrexy4ever@yahoo.com; 6 Faculty of Food Science and Fisheries, Universiti Malaysia Terengganu, 21030 Kuala Nerus, Terengganu, Malaysia

**Keywords:** proximate analysis, fatty acids, organoleptic assessment, boiling

## Abstract

This study evaluated the effects of cooking for different hydrothermal-treatment durations (10, 20, 30 and 40 min) on the proximate composition, amino acid profile, fatty acid composition and organoleptic parameters of filets of African catfish *Clarias gariepinus* (Burchell, 1822). Filets of the fish were prepared from market size African catfish with similar breeding history. Parameters of the processed filet under the different hydrothermal durations were also compared against a raw–unprocessed control group except during organoleptic analysis. The results obtained revealed a significant increase in protein, fat and ash content until the 30th minute of hydrothermal processing (*p* ≤ 0.05). Beyond this processing time, protein and fat significantly reduced while ash remains unaffected. The same trend was observed for most essential/non-essential amino acids isolated as well as the prominent saturated fatty acids, monounsaturated fatty acids and polyunsaturated fatty acids. In all, the raw control group consistently recorded the least values of nutritional components. The perception of assessors was, however, found to be similar (*p* ≥ 0.05) in terms of organoleptic parameters regardless of the duration of the processing time of the filets. It was concluded that cooking the African catfish filet using the hydrothermal method should not be extended beyond 30 min.

## 1. Introduction

Nutritionally, fish has been prided to be superior to meat in terms of quality protein (i.e., good amino acid profile), high mineral content and low saturated fatty acid [1]. Fish constitute a major component of the diet of both low and high-income earners and an important part of animal nutrition [2]. Therefore, the continuous increase in the human population and higher living standard have led to a corresponding increase in demand for fish and fish products [3]. This has resulted in the rapid development of aquaculture as a means of augmenting fish supply from capture fisheries. Among the fishes popularly cultured in West Africa and many parts of the world is the African catfish *Clarias gariepinus*. Its ability to grow rapidly at very high densities, its efficient feed conversion, hardiness to poor water conditions and tastiness of its flesh make this fish an excellent candidate for aquaculture [4,5].

However, for it to be fit for consumption, it has to be cooked just like most fishes [6]. Fish cooking can be done through boiling (i.e., hydrothermal treatment), grilling, roasting, baking, frying or smoking [7,8,9]. Cooking through this different heat treatment can help to improve the value of the fish by enhancing their flavor/taste as well as inactivate pathogenic microorganisms present [8]. However, as cooking improve the digestibility of fish, it also affects thermolabile compounds; hence, causing macro and micronutrients distortions [7]. Most the changes observed occur in the proximate composition, amino acid profile and fatty acid composition of the fish [10,11]. This is because these nutrients are sensitive to heat, light, oxygen, pH or a combination of some or all of these factors [6].

The effects of different cooking methods (such as boiling, frying, microwaving, etc.) on the nutritional characteristics of different fishes have been well documented in many previous studies [7,8,11]. However, the duration of the processing of the fishes in these studies was on the premise of the observation of filet tenderness without individually optimizing the different cooking methods. Since, heat treatment causes significant chemical and physical changes, which invariably improve or prejudice the fish’s nutritional value [7], it is imperative to optimize the duration of cooking in an attempt to preserve nutritional quality without jeopardizing consumer’s perception of the cooked fish. To our knowledge, no study has attempted to optimize individual heat treatment for the processing of fish. Neff et al. [12] had reported that hydrothermal treatment or boiling is a better option for cooking fish as it results in lower levels of less-favorable fatty acids (i.e., the saturated fatty acids) in contrast to other cooking methods. Hence, this study was designed to determine the optimal cooking duration (using hydrothermal treatment) that best improve the proximate composition, amino acid profile, fatty acid composition and organoleptic parameters of African catfish *C. gariepinus*.

## 2. Materials and Methods

**Figure 1:**
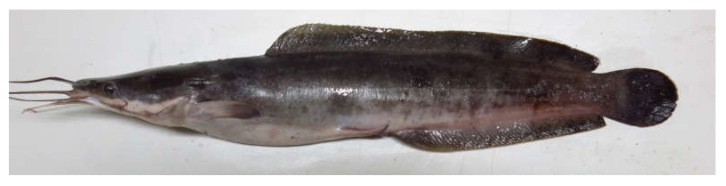
Specimen of African catfish *Clarias gariepinus.*

Thirty table-sized African catfish of about 1 kg by weight (Figure 1) from the same breeding history were collected from the Department of fisheries and aquaculture fish farm of the Federal University of Agriculture Makurdi. The collected fish were washed with tap water (to remove any adhering material) and placed on ice in insulated boxes for preservation. They were thereafter transported to the fish nutrition laboratory for further processing and experimentation. At the laboratory, the biometric parameters of the fish such as standard length (taken using measuring rule) and body weights (taken using an electronic weighing balance) were recorded. Fileting of the fish was then done by four skilled fish processors. The procedure for fileting was a modification of the method previously reported by Yenmak et al. [13]. The fish were cut from the top of the head, down the side behind the pectoral fins, and along the side of the dorsal fins with the aid of a sterilized sharp knife. The viscera organs, heads, fins and skeletons (frames) were then separated from filet and disposed of. Only the filet was used for further analysis.

To reduce the influence of the within-sample variation of the nutritional components, the method suggested by Neff, et al. [12] was adopted in this study. In brief, each strip of the African catfish filet was divided into five sections so that in each treatment in this study a portion of all the samples can be represented as sub-samples for experimentation and analysis. This means the filet was divided into five triplicate groups of about an equal weight (i.e., 600 ± 22 g comprising of three to four strips of filets) for the five different treatments designed for this current study. The first group was not processed hence used as the control (raw filet). The four remaining groups were hydrothermally processed in triplicate for 10, 20, 30, and 40 min in already boiling distilled water (100 °C) held in sterilized 15-liter metal cooking pots. No form of condiments or spices was added during the cooking process. After the different treatment durations, the boiling water was poured out and the fish held on a sieve for a few minutes to drain off excess water from them.

Triplicate samples of the cooked fish and the raw control were then nutritionally analyzed at the National Research Institute for Chemical Technology, Zaria. Analytical methods for crude protein, lipid, moisture, fiber and ash are as prescribed by AOAC [14] to determine the proximate composition for both the control and hydrothermally processed filet samples. Briefly, moisture content was measured by oven drying to constant weight, while crude protein content was measured by the Kjeldahl procedure. This procedure first determines the nitrogen percent (N) which is then converted to protein by multiplication by the factor of 6.25 (i.e., N × 6.25). Total lipid was determined using the Soxhlet extraction system. Ash was measured by gravimetrically in a furnace heated at 550 °C to a constant weight. Nitrogen free extract, however, was determined through the difference between the other components and 100. The amino acid profile on the other hand was determined using the method described by Spackman et al. [15]. For the fatty acid composition, the extracted fats were converted to free fatty acids by saponification. Identification/quantification of fatty acids was then achieved by gas chromatography-mass spectrophotometer, the former being resolved by elution times following the standard method by AOAC, [16].

Sensory evaluation was carried only for the hydrothermally processed samples leaving out the control. This was done by a panel of previously trained assessors (20 numbers) comprising of staff and students at the Federal University of Agriculture. Employing the 5-point hedonic scale described by Tobor [17], the samples were scored as follows; 5 = very good; 4 = good; 3 = fair; 2 = bad and 1 = worst. The characteristics of samples tested included their appearance, texture, flavor, palatability, and the general opinion of the filets been “well cooked”.

For data analysis, summary statistics of the parameters measured across the treatment were first obtained using the statistical software Minitab 14 for Windows. Thereafter, the data for nutritional profile and organoleptic analysis were tested for normality and homogeneity of variance before subjecting them to Analysis of Variance (ANOVA). Where significant differences occurred, means were separated using Fisher’s least significant difference at a significance level of p ≤ 0.05. However, when the assumptions of normality and homogeneity did not hold, data were alternatively analyzed using the Kruskal–Wallis nonparametric test.

## 3. Results and Discussion

The proximate composition of the African catfish filets as shown in Table 1 revealed that boiling for different durations resulted in a significant water loss in the treatment groups when compared to the raw control group (*p* ≤ 0.05). Processing the fish at a boiling temperature of 100 °C probably vaporized the moisture content of fish as the intensity seem to vary proportionally with duration. However, the moisture content was observed to be inversely related in value to lipid, protein and ash content. Generally, these nutritional compositions have earlier been reported to increase following application of different cooking methods [10,18,19]. The increase in the protein, fat, ash, as well as essential amino acids and fatty acids content of the fish samples after processing in this study, is attributed to condensation, as a result of dehydration. Similar to our school of thought, Garcia-Arias et al. [10] had hypothesized that the increases in protein, fat or ash contents could be explained by the reduction in the moisture content in the cooked fish samples. This is because as fish cooks, nutrients coagulate expending water from the muscle fibers, thereby changing the flesh from translucent to opaque in appearance [20]. Although there is a paucity of information on the optimization of heat processing for fish, however, the trend of observation in the current study is similar to previous studies that had compared different heat treatments for the processing of fish [8,21,22].

It is noteworthy that the protein and fat content of the African catfish appears to peak at the 30th minute and decreased thereafter. However, the mineral content of the fish seems not to be affected beyond this boiling optimum as the ash content was statistically the same thereafter. This affirms that prolonged duration destroys amino acids and fatty acids as observed in Table 2 and Table 3, respectively for the current study. The effect of prolonged hydrothermal processing on the denaturation of amino acids has been well documented in the processing of unconventional feed ingredients used in fish diet [23,24,25]. The trend of observation suggests that prolonged hydrothermal processing/boiling may substantially reduce ant-nutritional factors in the feed ingredient, but consequently denature protein and amino acids in the process, hence justifying the need for the optimization of the treatment duration [26,27]. Although many studies have shown conservation as well as variations (i.e., gain and losses) in amino acids with the application of different cooking methods for fish filet processing [8,9,11], this study has demonstrated that there is an optimal duration beyond which boiling can induce losses in protein nutritional quality. However, it is noteworthy that the values of sulfur-containing amino acids (i.e., methionine and cysteine) continuously reduced with duration, hence are suggested to be the most susceptible of the amino acids to heat treatment in this study. The most predominant isolated amino acids such a lysine, threonine, valine, aspartic and glutamic acids in this study were in tandem with values previously reported by Rosa et al. [7] for raw and cooked African catfish *C. gariepinus* using different heating methods.

One of the most important health benefits of the consumption of fish is the presence of a complex fatty acid profile in it. This includes saturated fatty acids (SFA), monounsaturated fatty acids (MUFA) and polyunsaturated fatty acids (PUFA) [8]. In this study, the fatty-acid contents followed a relative pattern of SFA > PUFA > MUFA. This pattern is different from the fatty acid signatures reported for rainbow trout, walleye pollock, smelt, canary rockfish, common carp, chinook and pink salmon [8,12,28]. Previous studies have earlier demonstrated the fact that fish fatty-acid content varies with many factors; prominent among which are species, season, fish size and geographic location to mention, but a few [29,30]. This probably explains the discrepancies in the fatty acid signatures in the different studies cited earlier. The most predominant SFAs isolated in the current study (Table 3) were lauric acid (12:0) and palmitic acid (16:0). Oleic acid (C18:1*n-9*) which was the most predominant of the MUFAs in the current study has been reported to be of exogenous origin and usually a reflection of the type of fish diet [31], hence, may differ even among the same species if fed different diet.

The eicosatrienoic acid (ETE, 20:3*n-3*), eicosapentaenoic acid (EPA, 20:5*n-3*) and docosahexaenoic acid (DHA, 22:6*n-3*) were the dominant PUFA isolated in both control and the treatment groups in this study. Fish long-chain *n-3* PUFA, especially EPA and DHA, are hypotriglyceridemic and are important in the prevention of human cardiovascular disease, diabetes, inflammatory diseases and neurological/neuropsychiatric disorders [32]. Because these fatty acids cannot be synthesized in amounts adequate for optimal health, they are identified as essential elements that must be incorporated in the human diet [33,34]. The observation of EPA+DHA content in this study for both raw and cooked African catfish is far above the recommended daily intake of 0.25 g as suggested by different agencies and authors [35,36]. Similarly, the PUFA: SAFA ratios in the current study are greater than >0.4 recommended by FAO [37].

Bhouri et al. [38] had earlier shown that the *n-3* fatty-acid content of farmed sea bream was substantially reduced after cooking using different methods. Kołakowska et al. [39] on the other hand observed that conventional cooking and microwaving resulted in approximately 10% change in the amount of PUFA (including EPA and DHA) of striped catfish filets, whereas the percentages of SFA and MUFA remain unchanged. Some studies have also reported significantly lower levels of EPA and DHA content after frying the fish [40,41]. On the other hand, some studies reported no effects of various un-optimized cooking methods on fish fatty acid composition [21,28]. Although Kołakowska and Bienkiewicz [42] had earlier hypothesized that heat treatment may cause an increase, decrease or not affect different fatty acids, the current study has however shown that hydrothermal treatment or boiling will must be done beyond 30 min before a significant reduction in most of the n-3 fatty acid is observed. This is an important observation from a human health perspective, as it suggests that boiling does not reduce the amount of beneficial fatty acids consumed compared to what is contained in a raw filet unless the optimum boiling duration is exceeded.

The sensory evaluation of the cooked samples based on the preference of the organoleptic panel showed no statistical difference (Table 4). This means duration seems not to affect the perception of consumers in terms of taste, flavor, and general acceptability of the filets been well cooked. An earlier study by Oparaku and Nwaka [22] reported that perceptions of assessors on organoleptic parameters vary with different processing/cooking methods with boiled fish products preferred more than solar-dried, oven-dried and smoked fish products. This observation and that of the current study may have affirmed the hypothesis of Neff et al. [12] that hydrothermal treatment or boiling is a better option for cooking fish than other cooking methods. However, future studies can focus on optimizing the duration of processing for other heat treatment methods to improve the nutritional content of the processed fish. Although this study optimized the cooking of African catfish at 30 min, this cannot be used as a rule of thumb for the processing of other fishes. Hence, the application of hydrothermal processing for other fish species also needs to be optimized in future studies to determine the best duration that improves nutritional quality and taste without denaturing essential nutrients. More so, there is a possibility that different heating sources and intensity of the hydrothermal processing method can result in variation in the result reported in this study using the optimized time reported. This can also be the focus of future researches.

## 4. Conclusions

This study has shown that the protein, fat, ash, essential amino acid and fatty acid content of the fish samples after processing significantly increased up to the 30th minute. However, the perception of assessors was not affected, as organoleptic parameters were similar regardless of the duration of the processing of the filets. Based on the observed differences in the nutritional content of the boiled fish in this study, it is concluded that the hydrothermal processing of African catfish should not exceed 30 min for nutritional conservation purposes.

## Figures and Tables

**Table 1:** Proximate composition of *Clarias gariepinus* hydrothermally cooked for different durations. Numbers are means ± standard errors.

Parameters	Raw	Time (minutes)				*p*-Value
		10	20	30	40	
Moisture	63.31 ± 0.03 ^a^	60.43 ± 0.24 ^b^	59.43 ± 0.12 ^c^	58.47 ± 0.25 ^d^	57.44 ± 0.10 ^e^	0.001
Ash	1.49 ± 0.06 ^d^	3.39 ± 0.06 ^c^	3.55 ± 0.03 ^b^	3.92 ± 0.02 ^a^	3.93 ± 0.04 ^a^	0.001
Fat	1.44 ± 0.01 ^d^	2.59 ± 0.13 ^c^	2.67 ± 0.05 ^c^	3.67 ± 0.00 ^a^	3.44 ± 0.12 ^b^	0.001
Fiber	1.68 ± 0.05 ^c^	2.07 ± 0.02 ^b^	2.17 ± 0.02 ^a^	2.09 ± 0.01 ^ab^	2.6 ± 0.01 ^b^	0.002
Protein	18.66 ± 0.10 ^e^	22.45 ± 0.12 ^d^	24.84 ± 0.05 ^b^	25.83 ± 0.04 ^a^	23.41 ± 0.06 ^c^	0.001
NFE	13.44 ± 0.22 ^a^	9.08 ± 0.42 ^b^	7.16 ± 0.15 ^c^	6.03 ± 0.30 ^d^	9.73 ± 0.24 ^d^	0.011

* NFE—nitrogen free extract, determined by difference. Means in the same row with different superscript (a-e) differ significantly (*p* ≤ 0.05).

**Table 2:** Amino acids composition (g/100 g protein) of *Clarias gariepinus* hydrothermally cooked for different durations. Numbers are means ± standard error.

Amino Acids	Raw	Time (Minutes)				*p*-Value
		10	20	30	40	
**EAA**						
Lysine	1.28 ± 0.01 ^d^	1.47 ± 0.03 ^c^	1.59 ± 0.01 ^c^	2.18 ± 0.02 ^a^	2.05 ± 0.02 ^b^	0.001
Histidine	0.11 ± 0.01 ^d^	0.35 ± 0.01 ^b^	0.42 ± 0.02 ^a^	0.43 ± 0.01 ^a^	0.25 ± 0.01 ^c^	0.001
Arginine	0.58 ± 0.01 ^d^	0.65 ± 0.01 ^c^	0.74 ± 0.04 ^b^	1.06 ± 0.02 ^a^	0.78 ± 0.02 ^b^	0.003
Threonine	1.08 ± 0.03 ^d^	1.86 ± 0.03 ^c^	1.92 ± 0.01 ^b^	2.07 ± 0.01 ^a^	1.95 ± 0.02 ^b^	0.002
Valine	0.71 ± 0.01 ^e^	1.18 ± 0.01 ^d^	1.43 ± 0.03 ^c^	1.98 ± 0.01 ^a^	1.88 ± 0.01 ^b^	0.001
Methionine	0.95 ± 0.02 ^a^	0.69 ± 0.02 ^b^	0.55 ± 0.01 ^c^	0.44 ± 0.01 ^d^	0.31 ± 0.02 ^e^	0.011
Isoleucine	0.72 ± 0.02 ^d^	1.23 ± 0.02 ^c^	1.46 ± 0.03 ^a^	1.47 ± 0.01 ^a^	1.31 ± 0.01 ^b^	0.001
Leucine	0.91 ± 0.02 ^d^	1.43 ± 0.02 ^c^	1.57 ± 0.04 ^b^	1.62 ± 0.02 ^a^	1.60 ± 0.04 ^a^	0.001
Phenylalanine	0.51 ± 0.01 ^e^	1.03 ± 0.01 ^d^	1.42 ± 0.02 ^c^	1.76 ± 0.01 ^a^	1.54 ± 0.02 ^b^	0.001
**NEAA**						
Aspartic acid	1.58 ± 0.01 ^c^	1.75 ± 0.01 ^b^	1.89 ± 0.02 ^a^	1.84 ± 0.01 ^a^	1.75 ± 0.03 ^b^	0.001
Serine	0.22 ± 0.01 ^d^	0.58 ± 0.01 ^c^	0.73 ± 0.01 ^b^	0.89 ± 0.01 ^a^	0.69 ± 0.03 ^b^	0.001
Glutamic acid	2.39 ± 0.02 ^d^	3.01 ± 0.02 ^c^	3.19 ± 0.01 ^b^	3.54 ± 0.02 ^a^	3.16 ± 0.01 ^b^	0.001
Proline	0.51 ± 0.01 ^c^	0.95 ± 0.01 ^b^	1.04 ± 0.01 ^b^	1.19 ± 0.01 ^a^	1.21 ± 0.02 ^a^	0.002
Glycine	1.10 ± 0.02 ^d^	1.58 ± 0.01 ^c^	1.69 ± 0.01 ^b^	1.97 ± 0.02 ^a^	1.71 ± 0.02 ^b^	0.001
Alanine	0.82 ± 0.02 ^d^	1.12 ± 0.02 ^c^	1.71 ± 0.02 ^b^	1.98 ± 0.01 ^a^	1.68 ± 0.01 ^b^	0.011
Cystine	1.25 ± 0.01 ^a^	1.19 ± 0.01 ^b^	1.18 ± 0.03 ^b^	1.08 ± 0.01 ^c^	0.95 ± 0.01 ^d^	0.001
Tyrosine	0.51 ± 0.16 ^c^	0.86 ± 0.05 ^b^	0.94 ± 0.01 ^a^	0.98 ± 0.01 ^a^	0.89 ± 0.01 ^b^	0.001

EAA—essential amino acids. NEAA—non-essential amino acids. Means in the same row with different superscript (a–e) differ significantly (*p* ≤ 0.05).

**Table 3:** Fatty-acid content of *Clarias gariepinus* (g/100 g of dry weight) hydrothermally cooked for different durations. Numbers are means ± standard error.

Fatty Acid	Raw	Time (Minutes)				*p*-Value
		10	20	30	40	
C12:0	1.85 ± 0.02 ^d^	1.97 ± 0.11 ^c^	2.01 ± 0.01 ^c^	2.22 ± 0.02 ^a^	2.14 ± 0.03 ^b^	0.003
C14:0	0.68 ± 0.02	0.69 ± 0.01	0.76 ± 0.01	0.77 ± 0.02	0.78 ± 0.03	0.233
C15:0	0.16 ± 0.04	0.17 ± 0.01	0.17 ± 0.02	0.17 ± 0.03	0.17 ± 0.01	0.081
C16:0	1.57 ± 0.02 ^d^	2.01 ± 0.05 ^c^	2.12 ± 0.10 ^b^	2.13 ± 0.01 ^ab^	2.15 ± 0.02 ^a^	0.001
C17:0	0.16 ± 0.03	0.18 ± 0.02	0.18 ± 0.02	0.21 ± 0.01	0.20 ± 0.01	0.090
C18:0	0.59 ± 0.06	0.67 ± 0.14	0.67 ± 0.03	0.71 ± 0.04	0.71 ± 0.11	0.211
C20:0	0.85 ± 0.01	0.86 ± 0.03	0.88 ± 0.01	0.89 ± 0.02	0.85 ± 0.02	0.156
C22:0	0.45 ± 0.01 ^c^	1.05 ± 0.01 ^b^	1.07 ± 0.02 ^b^	1.15 ± 0.04 ^a^	1.20 ± 0.01 ^a^	0.001
ΣSFA	6.31 ± 0.04 ^e^	7.60 ± 0.02 ^d^	7.86 ± 0.01 ^c^	8.25 ± 0.03 ^a^	8.20 ± 0.01 ^b^	0.001
C16:1 *n-7*	0.38 ± 0.04 ^d^	0.45 ± 0.02 ^c^	0.48 ± 0.01 ^b^	0.53 ± 0.01 ^a^	0.46 ± 0.03 ^b^	0.001
C18:1 *n-7*	0.14 ± 0.04	0.14 ± 0.02	0.15 ± 0.01	0.15 ± 0.03	0.15 ± 0.01	0.120
C18:1 *n-9*	0.81 ± 0.06	0.81 ± 0.03	0.82 ± 0.02	0.82 ± 0.05	0.81 ± 0.10	0.522
C20:1 *n-9*	0.55 ± 0.01 ^a^	0.50 ± 0.03 ^b^	0.49 ± 0.02 ^c^	0.45 ± 0.04 ^d^	0.42 ± 0.01 ^e^	0.001
ΣMUFA	1.88 ± 0.04 ^d^	1.90 ± 0.05 ^c^	1.94 ± 0.03 ^b^	2.01 ± 0.05 ^a^	1.84 ± 0.01 ^d^	0.001
C16:3 *n-3*	0.32 ± 0.01 ^c^	1.01 ± 0.02 ^b^	1.03 ± 0.03 ^b^	1.10 ± 0.10 ^a^	1.09 ± 0.01 ^a^	0.001
C16:4 *n-3*	0.01 ± 0.01 ^c^	0.01 ± 0.02 ^c^	0.09 ± 0.01 ^b^	0.12 ± 0.03 ^a^	0.08 ± 0.02 ^b^	0.001
C18:3 *n-3*	0.02 ± 0.01 ^d^	0.04 ± 0.01 ^c^	0.09 ± 0.02 ^b^	0.15 ± 0.03 ^a^	0.09 ± 0.02 ^b^	0.001
C18:4 *n-3*	0.21 ± 0.01 ^e^	0.30 ± 0.01 ^d^	0.47 ± 0.02 ^c^	0.71 ± 0.01 ^a^	0.62 ± 0.02 ^b^	0.001
C20:3 *n-3*	1.02 ± 0.04 ^d^	1.12 ± 0.02 ^c^	1.29 ± 0.31 ^b^	1.45 ± 0.50 ^a^	1.39 ± 0.21 ^b^	0.001
C20:4 *n-3*	0.06 ± 0.01	0.07 ± 0.03	0.07 ± 0.01	0.09 ± 0.02	0.06 ± 0.02	0.058
C20:5 *n-3*	1.58 ± 0.03 ^d^	1.83 ± 0.01 ^c^	2.14 ± 0.02 ^b^	2.43 ± 0.01 ^a^	2.12 ± 0.01 ^b^	0.002
C22:6 *n-3*	2.24 ± 0.01 ^d^	3.05 ± 0.04 ^c^	3.31 ± 0.02 ^b^	3.39 ± 0.04 ^a^	3.28 ± 0.01 ^b^	0.001
C18:2 *n-6*	0.32 ± 0.14 ^d^	0.46 ± 0.05 ^c^	0.49 ± 0.31 ^b^	0.52 ± 0.50 ^a^	0.49 ± 0.21 ^b^	0.001
C18:3 *n-6*	0.74 ± 0.02 ^e^	0.81 ± 0.05 ^d^	0.99 ± 0.03 ^c^	1.15 ± 0.05 ^a^	1.09 ± 0.01 ^b^	0.001
C20:4 *n-6*	0.15 ± 0.01	0.17 ± 0.03	0.17 ± 0.01	0.17 ± 0.02	0.17 ± 0.02	0.334
C22:4 *n-6*	0.16 ± 0.01	0.14 ± 0.04	0.14 ± 0.02	0.13 ± 0.04	0.12 ± 0.01	0.111
C22:5 *n-6*	0.11 ± 0.01 ^d^	0.14 ± 0.01 ^c^	0.19 ± 0.02 ^b^	0.22 ± 0.50 ^a^	0.19 ± 0.21 ^b^	0.001
EPA+DHA	3.82 ± 0.03 ^d^	4.88 ± 0.02 ^c^	5.45 ± 0.02 ^b^	5.82 ± 0.01 ^a^	5.40 ± 0.02 ^b^	0.001
ΣPUFA	6.93 ± 0.03 ^d^	8.27 ± 0.02 ^c^	9.51 ± 0.02 ^b^	10.25 ± 0.01 ^a^	9.86 ± 0.02 ^b^	0.001
PUFA:SFA	1.07 ± 0.05 ^d^	1.10 ± 0.03 ^c^	1.21 ± 0.01 ^b^	1.25 ± 0.03 ^a^	1.20 ± 0.02 ^b^	0.001

SFA—saturated fatty acids; MUFA—monounsaturated fatty acids; PUFA—polyunsaturated fatty acids; EPA—eicosapentaenoic acid (20:5 *n-3*); DHA—docosahexaenoic acid (22:6 *n-3*). Means in the same row with different superscripts (a–e) differ significantly (*p* ≤ 0.05).

**Table 4:** Organoleptic features of *Clarias gariepinus* hydrothermally cooked for different durations. Numbers are means ± standard errors.

Parameters	Time (Minutes)				*p*-Value
	10	20	30	40	
Flavor	3.17 ± 0.48	4.00 ± 0.52	4.33 ± 0.21	4.00 ± 0.37	0.252
Appearance	4.00 ± 0.63	3.67 ± 0.49	3.68 ± 0.33	2.50 ± 0.56	0.217
Texture	3.67 ± 0.56	4.17 ± 0.40	3.67 ± 0.42	3.33 ± 0.49	0.668
Palatability	2.07 ± 0.02	2.17 ± 0.02	2.09 ± 0.01	2.6 ± 0.01	0.374
Well cooked	2.67 ± 0.42	3.33 ± 0.33	3.50 ± 0.34	3.33 ± 0.67	0.597

Means in the same row do not differ significantly (*p* ≥ 0.05).
